# Contemporary narratives about asymmetries in responsibility in global agri-food value chains: the case of the Ecuadorian stakeholders in the banana value chain

**DOI:** 10.1007/s10460-022-10405-3

**Published:** 2022-12-08

**Authors:** Claudia Coral, Dagmar Mithöfer

**Affiliations:** 1 Department of Agricultural Economics, Agrifood Chain Management, Humboldt Universität zu Berlin, Invalidenstr. 42, 10115 Berlin, Germany; 2 Sustainable Land Use in Developing Countries, Leibniz Centre for Agricultural Landscape Research (ZALF), Eberswalder Straße 84, 15374 Müncheberg, Germany

**Keywords:** European green deal, Fair trade, Equity, Social justice, Maximum residue limits, Voluntary sustainability standards

## Abstract

Global concerns over environmental and social issues in agrifood value chains have increased and are reflected in a number of voluntary sustainability standards and regulatory initiatives. However, these initiatives are often based on poor knowledge of production realities, creating a disconnect between producing and consuming countries. Through narrative analysis, this paper reveals asymmetries in the responsibilities of the various actors participating in Ecuadorian banana value chains, providing clear problem- and solution-framings. Despite the broad range of actors interviewed, our analysis reveals convergence in two main narratives that reflect asymmetries amongst local actors in terms of their ability to participate, ability to maintain participation, and adaptation strategies in view of changing external factors. One narrative relates to sustainability adaptations, the cost of which is not shared among all value chain actors. This is combined with a downward trend in the price of bananas and the lack of a differentiated price to account for losses and costs arising from increasing sustainability standards. Another narrative reflects a value chain structure that threatens small-farm survival. It highlights the urgency of protecting small-farm activity by enforcing national regulation and developing new market segments/models that understand small-scale producer reality. Study results show that the multitude of standards is not seen as beneficial and that downstream actors rather wish for common minimum standards to reduce business costs. Compatibility between standards and local realities should be a priority for global sustainability standards adoption. Building on the problems and solution-framings of local value chain links, we give voice to local actors, and link their perceptions to existing literature and discursive politics while contributing to social transparency and addressing the democratic deficit in agrifood value chains.

## Introduction

Global consumer concerns over environmental and social issues in the agri-food sector have increased and are reflected in the increased spread of certification systems such as Fairtrade, as well as initiatives fostering direct linkages between producers and consumers (Glasbergen and Schouten 2015; Mithöfer et al. 2017a, b; Lambin and Thorlakson 2018). Voluntary sustainability standards (VSSs) are defined by various standard-setting organizations, which include nongovernmental organizations (NGOs) (e.g., Fairtrade International, WWF), the private sector (e.g., GLOBAL G.A.P.), governments (e.g., EU or US organic standards) and multi-stakeholder initiatives (Higgins and Richards 2019; Meemken et al. 2021). New partnerships between governments, private companies, and NGOs are reshaping global environmental governance (Lambin and Thorlakson 2018; Kalfagianni et al. 2020). VSSs active in the banana sector such as GLOBAL G.A.P., Rainforest Alliance, Organic, and Fairtrade are oriented to meet multiple and co-existing sustainability goals such as maintaining food safety, preventing deforestation, tackling poverty, empowering producers, and they employ a wide range of sustainability metrics. Meanwhile, less attention is paid to the quality of trading relationships within the value chain (Voora et al. 2022). Since Northern economic interests often lead these initiatives, the voices of local value chain links are rarely included when standards are defined (Meemken et al. 2021). The proliferation of VSSs is associated with processes of deregulation and re-regulation in which retailers and large brands have increasingly played a more significant role in governing value chains (Nelson and Tallontire 2014). In recent decades, competition between standards, as each tries to gain market share, has also increased, driving down price premiums in agri-food value chains (Lambin and Thorlakson 2018; Dietz and Grabs 2021).

At the heart of the European Green Deal, the Farm to Fork strategy establishes regulatory and non-regulatory initiatives to ensure that all foods placed on the EU market are sustainable (European Commission 2020a). These include commitments to make pesticide restrictions (Maximum Residue Limits, MRLs) more stringent based on health risks (European Commission 2020b). Since the EU is the second-largest importer of agri-food products globally (European Commission 2019), the strategy is likely to impact third countries significantly; for instance, total agricultural production volumes are projected to fall in some regions (Beckman et al. 2020). While the Commission has committed to evaluating immediate, mid- and long-term impacts in third countries when setting MRLs on the basis of import tolerances, this is still being discussed (Matthews 2022). The Farm to Fork strategy also offers producer and consumer countries the opportunity to establish the supportive environment necessary for companies along international value chains to operate more sustainably; for example, certification to VSSs. However, certification involves extra costs, typically covered by a combination of a price premium paid by consumers of certified products, taxpayers in countries that subsidize certification, and third-party organizations (e.g., NGOs) and their supporters (Meemken 2021). The challenge is that it is increasingly difficult to determine who bears the economic burden and who captures the economic, environmental, or social benefits (Meemken 2021). Decisions are often taken based on poor knowledge of production realities and contexts, creating a disconnect between consuming and producing countries. In reality, although fresh fruit and vegetable production systems are ‘transnationalized’ and ‘global’, production remains largely localized (Alandia et al. 2020; Graz 2021). Because VSSs and sustainability initiatives introduce global norms to local contexts and interact with existing public governance systems, studies are needed to better understand the social processes involved in standard-setting, coordination, and legitimation (Lambin and Thorlakson 2018; Johnson 2019). Several studies have recognised the asymmetric power geometries of VSSs and multi-stakeholder partnerships; however, little has been done to address the exclusion generated by these schemes in terms of participation and distribution of benefits (see Wilson and Jackson 2016; Higgins and Richards 2019; Johnson 2019).

The Ecuadorian banana value chain represents an example of a transnational and global value chain, producing 31% of global banana exports (FAO 2021a). Local actors in the Ecuadorian banana value chain have taken action against unfair trading practices. However, the market-driven economy, combined with oversupply and market power exercised by various value chain actors, is currently driving the chain’s dynamics.

Through narrative analysis, this paper aims to analyse the nuances of asymmetries in the responsibilities of the various actors participating in Ecuadorian banana value chains, providing clear problem- and solution-framings that reflect differentiated social needs. This article contributes to the debate on fairness and shared responsibility in global agrifood value chains by building on perspectives of the chain’s local links; hence, giving voice to local actors, contributing to social transparency, and addressing the democratic deficit in global agrifood value chain governance.

This study does not address the politics occurring between actors at the discursive level (Skillington 1997; Hajer and Versteeg 2005) but, following the approach of Scoones et al. (2019), it highlights how narratives and their underlying framings can support the goals of local and vulnerable actors by integrating their problem- and solution-framings into broader discursive politics.

The following section details the methodology of how we disentangle narratives from interviews with 54 value chain actors. The subsequent section presents the results of our analysis, identifying the constructs underlying two dominant narratives. These narratives and underlying framings show asymmetries in problem-definition, causes, diagnosis of problems, moral judgement, and suggestions of solutions. In the final section, we discuss the main conceptual constructs underlying the two dominant narratives in light of the existing literature.

## Methodology

### Case study

**Fig. 1:**
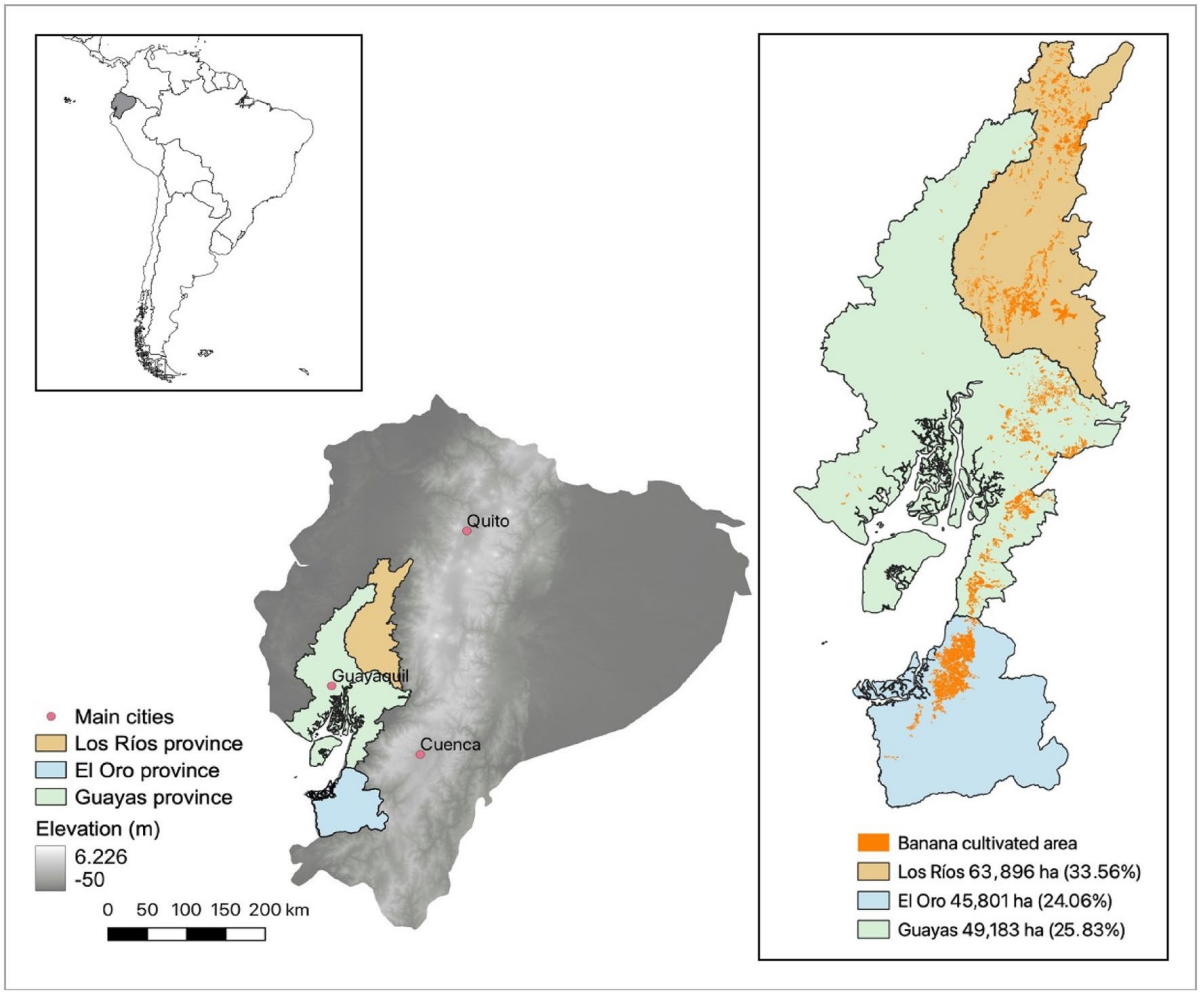
Estimated banana cultivation area in three study provinces that together constitute more than 80% of cultivated area in mainland Ecuador. *Source* Ecuadorian Ministry of Agriculture MAG – CGINA – DGGA, coordinated system: WGS 84 / UTM zone 17. Updated after the ESPAC 2019, published in INEC 2020)

From the 1950s and 1960s, coastal Ecuador experienced an export-oriented banana boom, becoming the largest exporter of bananas in the world, and accounting for 31% of the world’s banana exports (FAO 2021a). Bananas and plantain are Ecuador’s major agricultural export commodity, representing 41.8% of the country’s traditional non-oil–based exports (BCE 2021). In Ecuador, there are 8,851 producers, most of whom cultivate less than 30 hectares (61%) and less than 100 hectares (25%) (INEC 2020 cited in ACORBANEC 2021). More than 2.5 million people (around 17% of the total population) depend directly or indirectly on bananas to earn income (FAO 2021c). However, the total area under banana cultivation is very highly concentrated among a few medium- and large-scale producers, which relates to Ecuador’s historical land concentration and distribution trends (see Coral et al. 2021). Since the 1990s, the participation of independent national producers has been significant, and the importance of national exporters has increased. According to national survey data from 2020, around 160,000 hectares have been planted; more than 80% of these are cultivated in three coastal provinces: Los Ríos, Guayas, and El Oro (see Fig. 1) (INEC 2021). VSS-compliant production (certification with one or more schemes such as Organic, Fairtrade, Rainforest Alliance, GLOBAL G.A.P., and others) of bananas accounted for 42% of the total production in 2018 (Voora et al. 2022). Of the total volume of bananas produced in the country in 2020, 9.6% were organic; of the organic producers, 98% belong to consolidated associations that have managed to access international markets.^1^ Besides the traditional markets of Europe and the United States, which altogether accounted for 37% of the total export volumes, Ecuador also exports to other regions, including the Russian Federation, the Middle East, Asia, and Africa (CFN 2021; FAO 2021b). The vast majority of the fruit that is traded (about 90%) for export is sold on Free on Board (FOB) terms; this means that customers from abroad generally contract the shipping freight. The banana value chain involves several stages and stakeholders. Usually, national producers sell their product directly to the exporter or to an intermediary. The exporters often act as intermediaries between the producer and large transnational marketers. Some producer associations have managed to export their production directly. Large trading companies deliver the product to the importer, who does so in turn to a wholesaler, which is in charge of distributing to the ripening and trade companies who carry out distribution at the level of point of sale until it reaches the final consumer. In addition, a number of other stakeholders are part of the banana value chain, such as governmental and non-governmental; coordination; logistics and transport; and testing and certification actors.

The Ecuadorian banana export sector has been defined as highly politicized and highly regulated. For example, the Law to Control and Stimulate the Production and Commercialization of Bananas, Plantains and other Musaceae Destined for Exportation, created in 1997, prohibits any person, directly or through an intermediary, from paying the producer a value less than the minimum reference price set, and from using any mechanism or procedure to not comply with these provisions (FAO 2003). Additionally, the law restricts and regulates the establishment of new banana plantations (FAO 2003). In recent years, the Buenas Prácticas Agrícolas (BPA) national certification has been created to promote good agricultural practices, market access, and competitiveness for producers who receive a tax benefit for its adoption (MAGAP 2021). In 2021, BPA achieved equivalence with GLOBAL G.A.P. (MAGAP 2021). Furthermore, Ecuador has also formally sought mutual equivalence recognition for organic production with the EU as a strategy to alleviate the regulatory burden for local actors.^2^ These historical changes in the Ecuadorian banana value chain have being perceived in various ways and are reflected in contemporary narratives.

### Narrative analysis

Narratives are woven from threads of interviews, observations, and documents. They establish coherence across past, present, and as-yet-unrealized experiences (Riessman et al. 2008). The power of narratives lies in their simplicity, sense of urgency, and ability to enlist followers (Scoones et al. 2019). In this study, following Roe (1994, 1991) and Scoones et al. (2019), narratives are defined as storylines that start with a beginning (the definition of the problem), a middle (the elaboration of its implications), and an ending (the proposed solution). Narratives are underpinned by framings, defined as the conceptual constructs than inform and drive narratives (Druckman 2011; Entman 1993 cited in Scoones et al. 2019); frames serve as the underlying foundations on which narratives are expressed (Aukes et al. 2020). These underlying frames “*define problems* – determine what a causal agent is doing with what cost and benefits…; *diagnose causes* – identify the causes creating the problem; *make moral judgements* – evaluate causal agents and their effects; and *suggest remedies* – offer and justify treatments for the problems and predict their likely effects” (Entman 1993, p. 52). For the purpose of this study, narratives are analysed based on interviews with a broad range of participating actors.

#### Participants of the study, sampling method, and data analysis

Participants were sampled based on theoretical sampling. After the first data were collected and analysed, the resulting concepts provided the basis for subsequent data collection to explore these concepts further. We interviewed 54 actors (12 females and 42 males) from the following sectors: governmental and non-governmental; coordination; logistics and transport, including ports; production, including individual producers and representatives of producers’ organizations, cooperatives, and federations; exports; commercialization; certification; input and supply (see Table 1). Computer-mediated interviews took place from February to April 2021. Interviews lasted an average of one hour and were audio-recorded with participant permission granted. Interviews were confidential and anonymous to allow study participants to be comfortable sharing their narratives. All data were transcribed.

**Table 1:** List of study participants

Participants	Number of interviewees
Governmental	
National Phyto and Zoosanitary Regulation and Control Agency (Organic Direction)	1
Ecuadorian Institute for the Promotion of Exports and Investments	1
Ministry of Agriculture	1
NGOs and international cooperation agencies	
NGOs and foundations	2
International cooperation agencies	2
Banana coordination sector	
Ecuadorian Banana Cluster coordination	1
Banana logistics and transport sector, incl. private ports	
General managers private port terminals in Ecuador	2
Association of Private Port Terminals of Ecuador	1
Reefer, specialist international shipping company	1
Production and export	
Banana Marketing and Export Association (made up of 37 partners, the second largest export association in the country)	1
Transnational company dedicated to the production and commercialization of bananas	1
General Manager export company, < 200 containers per week	1
Regional Corporation of Ecuadorian ‘Bananeros’ (Union of medium and large-scale producers, representing 20% of production)	1
Regional Union of Peasant Organizations (11 affiliate associations, 300 small-scale producers, incl. agroforestry systems)	1
Presidents of small/medium-scale producer cooperatives and associations (also producers themselves)	9
National Federation of Banana Producers (mostly small and medium-scale producers)	1
General Manager/Presidents national producer and export company, < 2500–4000 ha	3
Banana producer, 100–500 ha	2
Banana producer, 50–100 ha	2
Banana producer, 10–50 ha	4
Small-scale producers, < 10 ha (conventional, Organic/SPP, Organic/Fairtrade, Rainforest Alliance, Global GAP certified)	3
Association of Banana Exporters of Ecuador (representing around 70% of Ecuador’s banana exports)	1
Certification	
International brand for certified products and certification	1
Executive director, certification label (farmer-owned certification system)	1
Representatives of certification bodies in Ecuador (mainly certifying organic— EU-Organic, USDA, Organic biologique Canada, GLOBAL G.A.P. + add-ons, Rainforest Alliance, SPP, Demeter)	4
Commercialization Europe	
European commercialization cooperative company (Organic, Fairtrade, SPP)	1
European commercialization association (fresh importer organic, Fairtrade, biodynamic bananas)	1
Agricultural inputs and supplies	
Corrugated cardboard packaging industry	1
Agricultural-inputs supply industry (agrochemicals)	1
Plastic inputs for the banana industry	2
Total	54

The narrative analysis was divided into two steps: first, an initial coding was developed inductively from the data. From the coding, we obtained the following main thematic areas: characteristics of the Ecuadorian banana sector; global links and markets; future challenges; past challenges; social and sustainability concepts; historical events/milestones; sector organization/coordination; organizations and actors’ characteristics; opportunities for the future; and gender. We did not undertake any quantitative assessment of the frequency of use of terms or phrases; however, the repetition of events and meaning assigned to events illustrate remarkable convergence on two main narratives reflecting actors’ experiences and the positions they intended to convey, hence reflecting a sense of ‘urgency’. We undertook a narrative analysis that centred on identifying the conceptual constructs that inform and drive narratives (Tables 2 and 3, following Entman 1993). These were linked to the existing literature and wider discursive politics (see Discussion section). Although narrative interview data is used as the main data resource for the analysis, the interview-narratives were factually cross-checked against additional resources such as policy documents, news, and reports. In the results section, we present the two narratives and exemplary quotes in narrative form to reflect how elements are interwoven, together composing a narrative. Quotations (raw data) are included for the sake of fidelity in, for instance, the expression of moral judgements. We then discuss the main conceptual constructs underlying the two dominant narratives (summarized in Tables 2 and 3), following Entman (1993), and these provide insight on asymmetries in problem definition, diagnosis of causes, and framings of solutions and consequences.

**Table 2:** Framing—conceptual constructs of narrative 1—asymmetries in responsibility among value chain actors

Problem definition	Causes and diagnosis	Responses/remedies/likely effects
Sustainability adaptations happen at a cost that is not shared among all value chain actors Loss in competitiveness against other producer countries with lower prices, thus influencing the total volume of bananas exported and market participation	Retailers’ low-price policy, aggressive pricing strategy Standard-setting organizations and the power given to them by retailers. Multiple, uncoordinated, and competing certification schemes coexist Other initiatives at the European level set both regulatory and non-regulatory standards; e.g., Maximum Residue Limits (MRLs) do not respond to reality of production in the tropics and business from the perspective of the local links in the value chain Customers do not always respond positively to higher fair prices, which might reflect the trend in consumer demand for low-priced bananas	The Ecuadorian Banana Cluster has reunited claims from producers, exporters, and banana industry associated sectors to establish ‘shared responsibility’ as a framework to distribute the costs related to sustainability standards among all the supply chain actors (producer–retailer–consumer) A task force composed of banana growers and exporters from seven Latin American countries Information and support regarding the application of agrochemical products and their regulation Setting minimum requirements at governmental and regional level Studies on the reality of banana production and understanding of the whole business from the perspective of the local links in the value chain Education and conscientization of consumers Advancing in research, creating alternatives with less chemical load to improve pest control, and aligning to organic; although organic production remains a small market segment, and import quota restrictions exist Greater tolerance in the establishment of aesthetic standards Building alternative trading routes to sell to more ‘tolerant’ markets that do not require certifications –potentially at the expense of lower prices, less reliable trading partners, and losses in local capability

*Source* Based on code scheme from transcribed interviews

**Table 3:** Conceptual constructs narrative 2—The structure of the chain threatens small-farm survival

Problem definition	Causes and diagnosis	Responses/remedies/likely effects
Challenges sustaining small-scale farming, due to competition between small producers and large estates, productivity, and problems of commercialization Smallholder producers are increasingly in competition with larger producers and transnational companies due to changes in the politics of the very same spaces (initiatives, mechanisms, and partnerships) that were once created to support them	‘Informality’—although there is a law that estimates a minimum price, the price is not always respected by informal intermediaries and exporters The market segment for organic and Fairtrade was mainly reserved for smallholder producers who benefited for many years; currently much room has been lost for Fairtrade international from small-scale producer associations since large plantations entered the Fairtrade market Although the organic market segment is still growing, it is still a small niche, and smaller producers are slowly losing this segment as large plantations have captured this lucrative market as well	Enforcement of national regulations A re-conceptualization of fairness and sustainability concepts Development of new market segments/models that understand small-scale producer reality Since 80% of banana production is from small producers, disappearance would mean a loss in development of the rural economy and its multiplier effects

*Source* Based on code scheme from transcribed interviews

## Results: narrative analysis

Narrative analysis reveals two main narratives and underlying framings. Narrative one is more reflective of the views of chain actors such as larger producers, exporters, and associated industries beyond the production stage (see Table 2), while narrative two is more reflective of the perceptions of small-scale producers and their associations (see Table 3). Tables 2 and 3 present a summary of conceptual constructs underlying both narratives; these are presented in narrative form in the next two sections. Analytical insights are discussed in light of the current discussion on fairness and shared responsibility in agrifood global value chains (see Discussion section.)

### Narrative one: shared responsibility

Ecuador is positioned as the main supplier of bananas to the EU. However, Ecuador has been losing competitiveness against other producer countries with lower prices, thus influencing the total volume of bananas exported. An increased proliferation of Voluntary Sustainability Standards (VSSs) and certification schemes has been observed by the value chain’s local actors; GLOBAL G.A.P., Fairtrade International, Rainforest Alliance, and organic are the most adopted in the Ecuadorian banana sector. The demand for bananas that comply with VSSs has been increasing, especially in Europe and the United States. The role of VSSs demanded by retailers has been criticized for going beyond their role as certifiers, *transforming itself into a regulatory entity, abusing the power given by the same supermarkets*. As participants perceive, *it is no longer a certificate, it is a marketing tool*. Market demands are drivers of change, as reflected in value chain actor testimonies:

…more than 50% of the hectares planted in Ecuador already have international certification of different types, ranging from GLOBAL G.A.P., Rainforest and even organic ones, out of conviction and by necessity also, obviously. To adapt to the markets, the trend… Europe and the United States do not represent more than 40% and the other 60% of the markets do not require certifications now. So, we are breaking paradigms in the first place but this is a matter of time before the rest will be added…, the problem is… that certain certifiers… want to go beyond the local regulations beyond what the Codex Alimentarius establishes...they have tried to implement a regulation, not discussed, nor socialized^3^…we have met with supermarkets, with authorities denouncing this publicly. (raw data 49, representative of banana marketing and export association)

For the banana crop, whose production systems have been identified as being environmentally and/or socially sensitive, multiple, uncoordinated, and competing certification schemes coexist.

…we have to enter into a variety of certifications today, starting with the BPA [national certification standard], which is a minimum requirement or GLOBAL G.A.P., but then they become stricter such as the Rainforest… how far are we going, how far are we allowing as Ecuadorians and producers that there are supermarkets or certifying companies that make their livelihood from this… and that want to convince supermarkets that there is a new certification and impose it on that country or that supplier; that should stop a little… sustainability is important, certifications are important because obviously that works in terms of the environment, that goes to protect our planet for the future of the next generation and we totally agree on that, it is important that there is a dignified and fair social treatment for workers and it is also important that a safe product is delivered to the whole world, with good nutritional qualities …but what happens is that today I do feel that there is a wrong concept of certifications, we cannot go with a banana that has 50 labels, Ecuador already has environmental parameters in its legislation, we have an environmental law... we also have a social security system and a fair salary... but all these investments are not valued in terms of price… I would say let’s stop certifications a bit, put a minimum standard at the European level, so let’s look for minimal requirements instead of thousands of certifications to come, the only thing they do is to contribute to the business of one more certification and that does not benefit the consumer, the supermarket, the producer nor the exporter... (raw data 5, representative of an association of banana exporters, and representative of national large-scale producer and export company)

Although an operator’s adoption of sustainability standards is voluntary, many state that it has become obligatory for market participation, resulting in huge operating costs and loads.

...you have these independent entities, or certifiers, who want to seek utopian sustainability, which is not yet adapted to the reality of the tropics. You need different types of conditions and different types of steps to be able to achieve it. There are many costs, low productivity, there is a lot of threat from pests, that is not understood, at the European level… So that’s the level of discussion that we’re having… (raw data 27, representative of large association of banana exporters)

Furthermore, several other initiatives at the European level set both regulatory and non-regulatory standards. Recently, the European Farm to Fork strategy has set the goal of making Europe the first climate-neutral continent by 2050. However, as a good number of participants in this study explain, the main worrying issue regarding the Green Deal is related to the MRLs established as part of a comprehensive policy for the protection of human health with regard to the use of pesticides in agri-food products. The elimination of some active ingredients that are used in conventional production has caused intense debates among actors in the banana value chain. As they express, these decisions place a serious weight on the cost of production and productivity itself, causing high agricultural losses. For instance, as participants in this study report, since the application of chlorpyrifos, a pesticide widely used in conventional banana production, was limited, losses at farm level have reached up to 30%. A number of participants claimed that these regulations reflect a lack of knowledge of tropical production*.* Additionally, the MRLs regulation would remove tools to protect production precisely when new tools are needed due to the Foc TR4 threat. The Fusarium wilt of bananas is caused by a soil-borne fungus that is highly aggressive towards Cavendish bananas. It was detected in South America in 2019 and, to date, there are no commercial banana cultivars with an effective resistance against it (see Cheng et al. 2019; Maymon et al. 2020). This point is illustrated in the testimony below.

The Green Deal has an aspect that is critical, which is the policies of the MRLs… in this process, the European Union is applying the reduction or almost elimination of more or less 900 molecules, that is, 900 products that are inputs for production, but in doing so it should be done according to the food codex and the rules of the World Trade Organization; however, it is going beyond that very fast, and it is removing tools to protect production precisely at a time when we need new tools due to the threat we have today from the Foc TR4, so there we have a debate in which we are working with the European Union to find ways because one thing is production in Europe and another is tropical production in our countries of America where we are subject to a large number of pests. (raw data 4, representative of coordination sector)

Actors in the banana export sector generally agree on sustainability and safety to protect the consumer and collaborators, but they call for a differentiated fair price that accounts for the losses incurred in the production and packaging process, as one participant explains.

If you ask me and you say ‘we want to remove all chemicals from conventional bananas and now this is the law’, what you are giving me to understand is ‘I want organic bananas, but I don’t want to pay for the additional cost it takes to make it organic’. Organic bananas cost more because by making it organic, your waste is greater and your production is lower, therefore, your costs are higher. Therefore, instead of selling it at such a price, you sell it $2–3 more expensive, then if you are going to apply the same rules to the conventional one and pay the same price, it is not justified… Now, what is going to happen is that the banana is going to be full of bug bites. You have to throw that fruit away because it no longer fits into the box [due to quality and aesthetic standards]’ (raw data 39, representative of a large national export company, also a producer)

Agricultural losses often happen due to supermarkets’ strict aesthetic standards, which cause at least 10% of losses for producers. In this regard, as the participant below claims, “*to what extent is the waste of food compatible with sustainability?*”.

… this is having a substantial decrease in production, this will also increase the losses, the rejections, then to what extent is the waste of food compatible with sustainability? In other words, they want no use of agrochemicals. Well, then tolerate the physical form more, right? don’t care so much about the physical aspects or presentation… (raw data 31, representative of large national banana producer company)

Participants in this study have suggested that to achieve better control of consumer health, agreements must be reached on alternatives that enable maintenance of aesthetic and quality standards and eventually tolerate banana imperfections when these do not affect the fruit’s flesh. As participants have stated, there are organic alternatives but higher input and labour costs.

A downward trend in the price of bananas began five years ago. Since 2018, local actors have organized, starting with an open letter opposing German retailers’ low-price politics. European retailers have been reducing their buying price on a year-to-year basis, but at the same time continuing to demand more investment in sustainability certifications; this situation is combined with increasing production costs, as explained by the participants of this study.

… the pressure of the buyers of bananas causes the drop in the international price of the fruit, and this also contrasted with an increase in the demands in international markets regarding the fruit as such. European supermarket chains are the ones that basically, as they are the largest buyers of bananas at the European level, set the minimum price of the box of bananas at the international level; this minimum price has not increased in recent years but rather decreased, and this is contrasted with the demands of the same supermarkets at a European level because supermarkets now require certifications such as Rainforest or organic, so it is not logical, it is not coherent that more certifications or requirements are required for export and that you want to pay a lower price… that has been a challenge that the Ecuadorian banana sector has faced in recent years…(raw data 1, representative of the governmental sector)

... the northern European market, but specifically Germany, on the one hand, demands a lot of sustainability, which requires a lot of investment; however, prices have systematically dropped during the last 15 years… despite the official minimum support price that exists in Ecuador. To give you an example, this year, the bunker is rising, which makes freight more expensive, which causes the price of plastic to rise, which causes the price of cardboard to rise, which makes price of fertilizer increase, but in supermarkets the price of a box of bananas fell in between 0.55 to 0.70 cents per box… but it is a very flawed market, as it is very competitive, I can say ‘no, I cannot afford it’, but another is going to come, [transnational companies] will come and they will say ‘yes’…But companies of our size cannot do it, so it is a difficult profession, very difficult. (raw data 39, representative of a large national export company, also producer)

Further, as producers have stated, they lack information and support regarding the application of agrochemical products and their regulation. They also point out a lack of studies on the reality of banana production and a lack of understanding of the whole business from the perspective of the local links in the value chain. Finally, they call for education and conscientization of consumers since they are also responsible for sustainable value chains. In other words, the critical issue is that sustainability adaptations happen at a cost that is not being shared among all value chain actors.

Recently, the Ecuadorian Banana Cluster has reunited claims from producers, exporters, and banana industry associated sectors to establish ‘shared responsibility’ as a framework to distribute the costs related to sustainability standards among all the supply chain actors (producer–retailer–consumer)*.*

… the banana sector has an economic, social, and environmental impact in the country; in that line, we have articulated a sustainability process that defends the environment that defends the social aspect, that defends the rights of workers because it cannot be sustainable if you do not have that focus in your long-term work plan, that is, it is not only an ideological, human, social issue as it should be, it is also the economic foundation that your industry can be competitive abroad… where a conflict occurs is that it involves a cost that has to be paid by the consumer because of a sense of shared responsibility. (raw data 4, representative of coordination sector)

Over the last year, the members of a task force composed of banana growers and exporters from seven Latin American countries (Colombia, Costa Rica, Dominican Republic, Ecuador, Guatemala, Honduras, and Panama) who altogether represent 65% of banana imports to the EU have denounced the abuse of market power exercised by a variety of actors in the value chain, such as retailers and standard-setting organizations.

Customers can play a decisive role; however, as the participants argue, customers do not always respond positively to higher fair prices, which might reflect the trend in consumer demand for low-priced bananas (see also Knowles 2019). Nevertheless, opportunities include advancing in research, creating alternatives with less chemical load to improve pest control, and aligning to organic.

… producers are interested in selling the fruit as organic because there are specific niches that pay very well for the fruit and are dedicated to that. The cost-benefit of banana production in Ecuador is now very, very tight, and that extra organic payment rewards them for all the effort they make, so organic certification is a benefit for them. (raw data 37, representative of medium-scale producer association)

However, organic production remains a small market segment, and there are import quota restrictions; in fact, some producers are already seeing the impacts of these restrictions.

… many people are already left with unsold, organic fruit. But with all this, with all these adjustments that are being made, people will continue to change towards organic. That is why we are worried, too, because obviously, the more supply there is, the prices will go down. (raw data 40, representative of national large-scale producer and export company)

… many organic producers have to sell as conventional because the market niche or the contract has already been closed or because they could not access that quota that the company gave them because in Ecuador, generally, the quota is handled by the transnationals, so the transnationals have their established quota of how many boxes they want to carry in organic, and if you do not have access to one of those quotas, it will simply be your turn, despite being organic, you will have to sell as conventional. That is really ... it can happen to you, and it has happened. (raw data 24, representative of producer sector)

Finally, one alternative that has been discussed, besides migrating to organic production, is that many producers would seek to sell to more ‘tolerant’ markets: so-called emergent markets with less stringent standards, but potentially at the expense of lower prices, less reliable trading partners, and losses in local capability, as one participant explains below.

... there are producers who will have to migrate or who have already migrated to organic or will have to sell to more tolerant markets. On this issue, the most tolerant markets are also the most defaulted on the issue of payments… there is high demand, that appears from the Middle East, Europe, China ... but also when demand drops, they pay $2 [per banana box] which is not even enough for production. (raw data 36, representative of a small and medium-scale producer association)

### Narrative two: the structure of the chain threatens small-farm survival

Smallholder producers face challenges sustaining small-scale farming, due to competitiveness, productivity, and commercialization problems. The root of these problems is a combination of a market-driven economy, overproduction, and power relations which are currently driving the dynamics of this global value chain.

The so-called ‘banana law’, establishes, among other things, minimum reference prices for a box of bananas, e.g., a minimum reference farm gate price and a Free on Board (FOB) price. However, some call the banana law *obsolete* and *irrational*, *a straitjacket,* *a law that generates transactional costs,* and *a law that does not allow growth in the planting area*. Furthermore, Ecuador is said to lose out to other countries that deliver at more competitive prices. On the other hand, other participants, representing small-scale producer cooperatives and associations claim that … *in the face of evident inequality, as we have in Ecuador, because there are many small farmers, in some way, they have to be protected with a law establishing limits… that regulates the small producer, the medium producer, and the exporter* (Raw data 24, representative of small-scale producer cooperatives and associations).

A situation of ‘informality’ has been reported. As explained by the participants, the term ‘informality’ refers to a widespread disrespect and lack of enforcement of the ‘banana law’, reducing protection of the smallholder producer.

…. We have, by law, to contract the fruit to export it, but that law is not enforced by, let’s say, the people who have to enforce it. So, everyone does whatever they want, there is a lot of — the right word is ‘informality’. Producers who sign contracts, when there are times in the year when the fruit [price] rises a lot, disrespect it [the contracts] and, instead of selling a box at the official price [reference price of 2021, $6.25 per box], they sell it for $9 or $10, as it is at the moment. And when things change, the other way around, when they [international buyers] don’t demand much fruit, companies disrespect contracts, they don’t pay us the official price and they pay us up to $2–3 per box. So, in both circumstances they pay justly for sinners, because there are producers who do respect and still are disrespected… what is happening right now? The exporters, also called ‘swallows’ because only show up in the good times, when the box is at $3, those swallow exporters double the purchase…They bring the fruit to Europe, to the Mediterranean, they arrive with that fruit to the United States, to the Middle East, bought at $3 plus export costs, let’s say $5 more on it, they arrive with that fruit at $8 to the foreign market, to the port, and those who have signed a contract, who cannot pay less than $6.30 arrive with that fruit at $6, plus $5 export costs, at $11, to the same markets. Then the ‘swallow’ buyers rip the formal exporters to pieces. (raw data 23, small-scale producer).

This ‘informality’ was particularly perceived and reported during the 2020 COVID-19 crisis, as narrated by this powerful testimony of a smallholder producer.

… Even though everything was sold, it was sold for dismal prices. They [exporters] did not respect the contracts, nor the prices. Many people were scammed, many people are indebted, we are just wanting to catch up with the debts… prices reached US$2–2.50 for a box of 43 and up to 45 pounds of premium, premium fruit… because, I’m honest, many weeks I chose to only take out what was for the week, what I could send and what I had commercialized [contracted], and the rest, I preferred to lose, or give it away or return it to the farm, minced, because it is the fruit of my work, my effort. Why am I going to give it to them and, we, a third-world country, wanting to get ahead, why do we have to feed rich countries, sell them bananas at US$2.50 and they eat a good quality banana, by far, that has many powers, nutrients and everything, which I know is one of the fruits that is accessible to the general population, in all markets? So, how can it be possible that we have to sell, the poor country, Ecuador, giving away the fruit of our labour, the bananas, and bringing misery to the country? And that is what the exporters have done. But the intermediaries and the exporters are doing well, very well and have grown overnight…. (raw data19, small-scale producer)

Until one decade ago, the market segment for organic and Fairtrade was mainly reserved for smallholder producers who benefited for many years, contributing to better life quality for producers, workers, and their families. The producer association and cooperatives investment of the Fairtrade Premium was mainly dedicated to improving household conditions, health access, education, and enhancing on-farm productivity and lowering banana production costs, water protection, reforestation, and recently COVID-19–related costs, as our participants narrate. As participants explain, small producer associations grew as Fairtrade grew as well, the growth of the one depending on the growth of the other.

... Fairtrade, for me or for our partners, is very important, and we respect it a lot because many producers have come out of the banana crisis in Ecuador, too big, too strong, many about to lose their farms, about to lose their livelihood, which we have been working on, not from now on, but from our parents, our grandparents, who have been banana growers. We have great respect for Fairtrade and for Fairtrade banana consumers because it is a help, it is a tool that was given to the Ecuadorian banana, especially Ecuadorian, because here it was the biggest problem of inequality… because it is not only a help for the producer... It is also a tool to offer a better quality of life to workers, our family, our children, to offer our children studies. For us, as producers, Fairtrade has given us a lot, a lot ... a lot of tools to be able to continue fighting, to continue winning the battle against this unequal struggle... (raw data 36, small-scale producer)

However, the concept as conceived in the 1980s has changed; it has “got bigger”, since private plantations also entered the Fairtrade International^4^ system as an opportunity to reform labour practices in the plantation sector.

… about 10 years ago, smallholder producer associations … were the ones that commercialized a very high percentage of Fairtrade bananas; nowadays, the large companies that dominate bananas, [name of transnational company] for example, once again have the same percentage of Fairtrade bananas that they have in the conventional market. So, in that case, Fairtrade has failed; the fair trade approach is to change commercial relations for small producers in an unfavourable situation… multinationals impose the rules, so in the end, the same unfavourable rules apply to the small ones in the same segment that had to save them, so there is not much to do… the big ones are choosing the most efficient, the cheapest, they put them in competition with the plantations, and they are going to put them out of market… the fresh banana market is a market of price and distribution capacity, a small company cannot, I think it is too harsh. You know that if you create a mechanism in which you put multinational companies and plantations with small ones in competition, the small ones will be driven out of market…(raw data 7, representative of the commercialization sector)

In fact, although Fairtrade International was initially addressed to smallholder farmers, currently, it can be adopted by both smallholder organizations and companies/plantations. Small-scale producers report insufficient market demand from European Fairtrade, and a lot of room has been lost for Fairtrade from associations. As perceived by Fairtrade participants, the reasons for the preference of bigger producer companies are traceability and quality issues, and homogeneity of production to meet demand. Besides, smallholder producers represent higher logistics costs. Hence, smallholder producers are increasingly being put in competition with larger producers and transnational companies due to changes in the politics of the very same spaces (initiatives, mechanisms, and partnerships) that were once created to support them and that were able to understand their struggles.

.... Many times, an importer puts conditions on us, he says ‘ok, I want organic, Fairtrade fruit, but I don’t want many codes. No, I don’t want to deal with many producers, I want to deal with only one’ and there they put us in a difficult situation because we are not one. Because we are not big, we are small and, being small, we are many. We are not able to market the fruit of a single producer, so we need to work collectively, in strength, in unity. And that has been one of the great limitations that we have been observing in recent years… we are at great risk, a strong risk of disappearing. (raw data 46, representative of small-scale producer cooperatives and associations)

Normally, the little ones represent a higher logistics cost, but you have higher quality, the big ones give you the volume when you need to make volume, or combine different brands in a process because you have one brand that only takes the large fruit and another the small fruit, so a small farm cannot do it. (raw data 39, representative of a large national export company, also producer)

Although the organic market segment is still growing, it is still a small niche, and smaller producers are slowly losing this segment. Participants report that this is already resulting in the displacement of organic producers and a greater supply of conventional bananas, since they are forced to commercialize their production as conventional.

… conventional Fairtrade banana producers were left without Fairtrade. They had to sell conventional, but no longer Fairtrade… organic Fairtrade did not buy from them, for consecutive weeks, and they were forced also to go conventional to sell their bananas. So, what is going to cause this? The displacement of hundreds of small producers… And what does it imply? That there will be a greater supply of conventional. (raw data 42, representative of small-scale producer cooperatives and associations)

Hence, the big question for small- and medium-holder producers has been how to stay in the market and what the alternatives are.

Stay in this market. That, the truth, is super complex, because now there are many large producers, many ... who have lower prices, who are dedicated to precision agriculture, to have much lower costs per box, and a small producer, already aged, because the population of producers is also ageing, so there are also many elderly people, who no longer handle technology… (raw data 17, representative of small-scale producer cooperatives and associations)

As our participants report, the disappearance of the ‘small ones’ would seriously impact the family-farm based economy. Small-scale producers are mainly organized in smallholder producer associations and cooperatives, many of which have managed to export directly. Beyond the loss of family farms, this would also mean a loss of the multiplier effects on farm diversity, associated national industries, spatially dispersed business opportunities in the rural economy, and employment.

Ecuador has a very different marketing structure than other countries. In Ecuador, there are 8,000 producers. In other countries, there are 50 …. they live in different cities of Ecuador and this energizes local economies. And since there are so many producers, there are also so many hundreds of input sales, marketing, import, and technical consulting companies. So sometimes, some value chain links do not realize that what they want to do is that there are no longer 7,000 producers, but that there are only 100, and they manage everything. But then there would also be fewer of these product importing companies because they themselves become the importers, they themselves become the fumigation companies, they themselves become the technical and commercial advisers and everything. So that’s the long-term problem, and maybe not everyone is seeing it. (raw data 15, small-scale producer).

…. We here have an advantage that 80% of our production is from small producers… that is an advantage because if we had it well regulated, the money would not fall into the hands of old men from 15 companies, but would fall to a good part of the country’s people. But as we go, the small producers tend to disappear and the worst thing is that in the country, sometimes the business is so bad, but [nevertheless] there are more and more hectares planted… and every day there are more large producers and every year, it is already 20 years that every year we export more. So, there is something wrong. (raw data 23, small-scale producer)

In the last decade, alternative labels, concepts, and certifications have emerged, such as the Small Producers’ Symbol (SPP), a Fair Trade certification system that belongs to Small Producers’ Organizations, which entered the international market in 2011. This initiative was supported by a number of commercialization cooperatives and producer cooperatives; however, challenges also exist.

… our prices are generally higher so people put us aside and say ‘well, if I can buy a concept for 10, why am I going to pay 11 or 12?’ Especially those companies that are not very committed or companies that find themselves in situations of a lot of competition say, ‘Well, the [name of a Fair Trade label] looks very nice but, well, I’m going with the cheap one’. So, the price kills the concept. (raw data 26, representative of certification sector)

Biodynamic agricultural standards have also been mentioned as an alternative. However, most notably, our participants call for a re-conceptualization of fairness and sustainability concepts and the development of new markets.

[Fair Trade] has enormous potential, and it has shown it… the important thing is that we always have to see who it is serving. It is a tool, and as such, it is not good or bad; that is, it depends a lot. It may be, in general terms, better than normal trade, but ultimately it depends on what model it serves, what it aspires to... Just as a term alone, for us it is not enough. Is it a fair trade that, finally, continues to build on the same canons of concentration of power in a few hands and the enrichment of some people, private companies? What really merits, for us, is the original philosophy of fair trade, which is closely linked to those processes of inclusion and a democratic struggle for a democratic economy and care for the environment. (raw 26, representative of certification sector)

So, the truth is that, for example, promoting agroforestry bananas to the consumer would have to develop a market in which you tell the consumer, ‘that’s it, the agroforestry banana is not at €1.59, it is at €2.50 [per kilo], period’; you would consume a totally different product. So that is something that could be promoted, but I would say that today it is something that does not exist in the market. I think that the actors in the chain do not want to promote that, they have already managed to impose on the consumer that the maximum is organic, coming from wherever it comes… if an organic actor from a store says ‘well the large plantation controls better their pests and does not have pesticide residues’, many people will stay with that argument and in the end the Latin American problem of competition between small producers, large estates, land concentration, in Ecuador water concentration^5^ won’t change. (raw data 7, representative of commercialization sector)

## Discussion

Although a broad range of actors were interviewed, our analysis reveals convergence in two main narratives and underlying framings. The first narrative captures the views of large producers and exporters and their representatives, while the second narrative reflects the views of smallholder producers and their associations (see Tables 2 and 3). Both narratives point to asymmetries in terms of unequal trading practices and a concentration of power for the benefit of a few actors. However, they diverge in their causal-agent mechanism, and their problem- and solution-framing. As observed in this study, beyond solutions and suggestions of remedies, these narratives also reveal actors’ resilience and adaptation strategies in view of changing external factors.

The first narrative deals with the fact that sustainability adaptations happen at a cost that is not shared among all value chain actors. This is combined with a downward trend in the price of bananas and the lack of a differentiated, fair price to account for losses and costs arising from increasing sustainability, aesthetic, and quality standards. The role of VSSs and retailers’ pricing policies have been central to the discussion (see Table 2). From the producer’s point of view, although the adoption of sustainability standards is voluntary, it has become obligatory for market participation. Although interviewees see VSSs as contributing to reducing the detrimental impacts of agriculture (confirming the findings of Smith et al. 2019; Sellare et al. 2020), they are insufficient to ensure food system sustainability at the production level as the prices offered do not cover certification costs. Hence, VSSs are not perceived as ‘fair’ or advancing equity objectives between upstream and downstream actors (see also Meemken et al. 2021). Similarly, VSSs in the global coffee industry show very little effectiveness in promoting holistic transitions toward more sustainable modes of production due to the paradox of being embedded in traditional markets and financing mechanisms (Dietz and Grabs 2021).

Although participants agree on the transformational capacity of VSSs, they find their role in recent years debatable. VSS-setting organizations are assumed to have multi-stakeholder governance structures that incorporate diverse actors’ interests; this is not the perception of local actors in practice. As observed by representatives of the banana export, production, and coordination sectors, although they participate in meetings with European supermarkets, standard-setting organizations, and authorities, the decision-making process often yields outcomes that are not ‘discussed’ and not ‘socialized’. This perception confirms results from Bennett (2017) who shows that, although producer groups are assumed to participate in governance, their perspectives often do not influence policy outcomes. Similarly, as observed in the palm oil industry of Ecuador, this uneven consensus reinforces and perpetuates existing patterns of exclusion (Johnson 2019). Effectively including producers in VSSs governance is critical to improving the democratic deficit at the international level (Bennett 2017). Further, the multitude of standards is not seen as beneficial and local links in the value chain rather wish for common minimum standards to reduce the costs of doing business and for greater tolerance in aesthetic standards, which would result in less agricultural loss and food waste. One step in this direction has been taken in recent years with the introduction of the Buenas Prácticas Agrícolas (BPA), a national certification created to promote good agricultural practices, which in 2021 achieved equivalence with GLOBAL G.A.P.

The European Farm to Fork strategy aims to ensure sustainable agricultural produce through regulatory and non-regulatory means (European Commission 2020a). However, in the last decade, international parties have done little to support developing countries; for instance, in the development of an enforcement and regulatory framework related to pesticide application (Handford et al. 2015). Hence, disharmonized standards act as a technical barrier to trade with additional high compliance costs for producers (Handford et al. 2015; Hejazi et al. 2022). Local actors expect the Farm to Fork strategy to significantly impact the Ecuadorian banana value chain. They express worry over the establishment of Maximum Residue Limits (MRLs) and the elimination of some pest control measures causing agricultural losses and lower productivity. As found in this study, before achieving the goals established by the strategy, several barriers must be overcome, such as a lack of information and support to the producer regarding the application of agrochemical products and their regulation and research on alternative supplies. Local actors further perceive downstream actors to lack knowledge of the reality of banana production; they additionally see a need for education and conscientization of consumers. Similarly, as observed by Matthew (2022), to achieve policy coherence for development, import standards should guarantee a coherent transition period to develop alternatives, ensure resources to support farmers adapting their production practices, and ensure full impact assessments and participation mechanisms like direct consultation and partnerships with exporting countries. Although there are not yet studies for EU trade partners in Latin America, in Spain, and all across Europe, farmers see a very unbalanced Farm to Fork strategy, claiming that the strategy lacks the right orientation (Tertsch 2020). For instance, Nes and Ciaian (2022) observe that alterations to marketing standards within the European Farm to Fork Strategy, for instance, might have food waste implications for non-compliant products, thereby negatively impacting the environment. As participants of this study claim, *to what extent is the waste of food compatible with sustainability?* However, as other studies suggest, in the absence of public standards, retailers may impose private standards that may be more costly for producers (Nes and Ciaian 2022). Similarly, European Farmers and European Agri-cooperatives (Copa-Cogeca 2021) argue it is necessary to assess the strategy’s impact on production capacity, competitiveness, consumer prices and ultimately on food security.

A critical fact is that increasing demands on producers represent a cost that is not reflected in the conventional price of bananas. Retail prices for bananas have been continually falling. The drop in import prices in the European Union is unprecedented (ACORBANEC 2021 based on CIRAD data; see also ABNB 2019; Wood 2021). In 2015, the Bureau for Appraisal of Social Impacts for Citizen Information (BASIC) estimated that, in the case of Ecuador, retailers earned around 42% of the total value of bananas while producers and workers only captured 7% each (BASIC 2015). However, internationally it has been difficult to establish the influence of agricultural traders on agricultural markets and food prices, often due to due to barriers in access to information (Salerno 2017). Customer education can play a major transformative role; however, customers do not always respond positively to higher fair prices, which might reflect the trend in consumer demand for low-priced bananas, as argued by study participants. In fact, consumers’ understanding of the multiple sustainability labels creates confusion and is a big challenge (Nes and Ciaian 2022).

The ‘shared responsibility’ concept proposed by a cluster of Ecuadorian actors represents a rapprochement between producer and exporter but also producer countries and import countries, recognizing the increasing responsibility of VSSs and retailers but also consumers through a constant process of dialogue. The World Economic Forum’s Global Agenda Council on Human Rights has defined shared responsibility as “a collective response in which global and local businesses, governments, international and philanthropic organizations devise collective solutions and share the financial cost of addressing the most entrenched human rights problems in global supply chains” (World Economic Forum 2015, p. 13). This shared responsibility involves assessing real costs and commitments, recognizing that neither companies nor governments alone can underwrite all of these costs, and generating cooperative approaches based on equitable responsibility-sharing for action among the key stakeholders (World Economic Forum 2015).

One adaptation strategy mentioned by study participants has been an alignment to organic. Historical data reveal a lower price risk for Ecuadorian organic bananas, compared with conventional bananas, even assuming a reduction in productivity of 35% for organic compared with conventional (Castro et al. 2015). Nevertheless, organic production remains a small market segment with import quota restrictions. Beyond organic production, an opportunity has emerged from the diversification of markets and growth in markets such as Russia, the Middle East, Eastern Europe, Africa, and the Eurasian zone, which currently demand mainly conventional uncertified product; however, often at a lower price. Hence, on the one hand, demand for Ecuadorian bananas from emerging and alternative markets with less stringent VSS requirements poses options for those with difficulties in meeting VSS requirements. On the other hand, selling to these markets is associated with lower prices and often less reliable buyers, as participants state. In the long run, a strategy of selling to less stringent VSS-requirement markets may imply losses on upstream actors’ capabilities, as shown for timber and cassava by Kaplinsky et al. (2011). The emergence of alternative trade routes may also contribute to circumventing and undermining the control of traditionally dominant firms. In our case, the multinationals along the chains serving the European and American markets, similarly to evidence from Thiers’ (2019) analysis of the effects of side-selling by contract farmers in the Philippine banana export sector, show this to result in the emergence of alternative trade routes at the loss of control by traditionally dominant firms.

The second narrative highlights a value chain structure that threatens small-farm survival. It mainly reflects the urgency of rescuing the market spaces given to small-scale producers and the need to protect their activity and fight informality by enforcing national regulations. Smallholder producers face difficulties sustaining small-scale farming due to competitiveness, productivity, natural hazards (safety and security), and commercialization concerns. The root of these concerns is a market-driven economy, combined with oversupply and market power exercised by various actors in the value chain. Related to the narrative framings, the Fair Trade discussion comes into play when evaluating causal agents and their effects (see Table 3). Although Fair Trade and the organic movements were originally characterized by small local operations, as participants in this study have stated, large plantations have captured this lucrative market. Historically the Fair Trade and the organic movements have been highly appreciated by smallholder farmer associations and cooperatives, allowing them to grow, as narrated by study participants. This growth also implies increasing organizational independence and auditability (Loconto et al. 2021). However, Fairtrade labelling initiatives and producer groups were sharply divided among themselves and between each other over plantations entering the Fair Trade market, resulting in the creation of new alternative labels, concepts, and standards, such as standards for hired labour (Moore 2004; Murray and Raynolds 2000; Smith 2010; Jaffee and Howard 2010; Harris 2021). Ever since small-scale farmers have competed against large banana-producing companies and plantations, their share of demand for Fairtrade-certified products has diminished. As our study participants explain, this is attributed to buyers’ preference for dealing with larger actors due to traceability and quality issues, and homogeneity of production to meet demand; smallholder producers also represent higher logistics costs. Fair Trade organization arguments in favour of the certification of large and smallholder producers were based on market concerns that plantations were needed to satisfy demand and civic/equity concerns that agricultural workers are amongst the world’s most disadvantaged populations and deserve equitable treatment (Raynolds 2017). However, it has been argued that the decision reflected the power and market priorities of dominant national labelling initiatives seeking to maximize labelled volumes, mainstream market sales, and the power of transnational corporations (Raynolds 2017). As analysed by Meemken et al. (2021), supply has grown faster than demand for many certified crops at a global scale. Estimates show that, given multinational companies’ increasing commitment to sourcing larger quantities of produce from sustainably certified production, the gap between supply and demand might tighten in the future (Meemken et al. 2021). In the case of Fairtrade-certified coffee, declining real prices reveal that the scheme was too closely tied to the same price-setting markets that the Fair Trade principles initially challenged (Bacon 2010). Furthermore, other studies show that price premia erode over time due to the market entry of additional actors (Molenaar et al. 2016; Naegele 2020). As observed in the case of Fair Trade coffee, the benefit to farmers might be overestimated when we take into account the output that is certified but not sold as Fair Trade (de Janvry et al. 2015).

Similarly, although smallholder producers have historically played a critical role in the production of organic bananas, recent changes in global organic market trends are having larger implications for smallholder farmers. As reported in this study, large plantations have captured this market. This phenomenon was first observed in the 2000s, when corporations significantly increased the scale of their operations, undermining the organic movement’s progressive social and environmental foundations (Murray and Raynolds 2000; Jaffee and Howard 2010). In Ecuador, this trend started recently, as our participants reflect; however, organic production remains a small market segment. Similarly, global trade growth in organic agrifood commodities has raised international competition and buyers’ quality expectations, displacing Dominican organic smallholder producers (Raynolds 2008).

Wilson and Jackson (2016) explore the interconnections between production and consumption and the historical and (post)colonial nature of food moralities. Their study suggests that despite the good intentions of those who promote sustainability standards, such policies better respond to the interests of (ethically ‘responsible’) consumers located in the Global North than to actors located in the Global South who are positioned primarily as the ‘ethically-demanding’ recipients of the intended and unintended consequences of such policies (Wilson and Jackson 2016). Similarly, Mutersbaugh and Lyon (2010) illustrate the many ways in which efforts to set up so-called ‘ethical commodity’ networks (those for which a significant share of value is rooted in ethical qualities that are in turn based upon publicly accepted standards) are undermined by historical circumstances and unequal power relations.

Although it has been stated that, globally, standards currently affect only a small number of farmers (Meemken et al. 2021), in the case of the Ecuadorian banana value chain, the transformative power of global demand is significant. However, as we can observe, strategies have been taken by actors in response to the increasing market power of downstream actors in the main traditional markets; for example, organisation in a cluster to denounce unfair trading practices, and building alternative trading routes to less stringent markets. In fact, the analysis of power in global value chains has shifted from focusing on buyer power to how key suppliers have established more powerful positions by following paths and strategies that create value and retain it (Dallas et al. 2019). Nevertheless, as our study participants have stated, the rejection of standards, for instance, also introduces a risk of exclusion, for example, a risk of losing competitiveness or being driven out of a market, as is already occurring in the Ecuadorian banana value chain. In the context of multi-stakeholder initiatives for sustainable palm oil and South-South trade, Higgins and Richards (2019) argue that standards and sustainability schemes generated towards markets in the Global South need to address the exclusion caused by these schemes. In the Ecuadorian case, the disappearance of the ‘small ones’, as our participants refer to them, would seriously impact the family-farm based economy because, of the 8851 Ecuadorian producers, more than 60% hold less than 30 hectares. They are mainly organized in small-scale producer associations and cooperatives, many of which have managed to export directly. Beyond the loss of the family farms, this would also mean a loss in multiplier effects on farm diversity, associated national industries, spatially dispersed business opportunities in the rural economy, and employment. As study participants have stated, a re-conceptualization of fairness and sustainability concepts is needed, as is the development of new market segments/models that respond to the reality of production in the tropics and business from the perspective of the local links in the value chain.

Methodologically, we highlight how narratives and their underlying framings can support the goals of local and vulnerable actors by identifying actors’ problem- and solution-framings. We interviewed a wide range of local value chain actors. However, one caveat of this study is that agricultural workers have not been interviewed due to the complex health situation and due to farm access restrictions and sensitivities during the data collection period. Hence, more research must be done to understand the perceptions of this group of actors. For future research, as Kano et al. (2020) conclude, at the micro-level, we need to pay greater attention to individual behaviour and the motivations of value chain actors and the ways in which these characteristics play out as multinational enterprises expand their value chains across geographies. Moreover, as Pegler (2015) suggests, it is necessary to compare the ‘logic’ (e.g., maximization and efficiency) driving global value chains with the ‘logic’ of the local actors at the beginning of the chains. Due to the diversity of production systems, ecosystems, and historical sociological contexts, compatibility between standards and local realities (‘institutional fit’) should be a priority for global sustainability standards adoption (Mithöfer et al. 2017a, b; Johnson 2019).

## Conclusions

Building on the underlying foundations on which narratives of local value chain links are expressed, this paper gives voice to local actors. It highlights differentiation in the social needs of important Ecuadorian banana value chain local links, forming the basis for reconciling divergent views of sustainability. The analysis shows asymmetries in perceptions of the status quo of shared responsibility, as well as asymmetries between large and small actors at the Ecuadorian end of the global banana value chain. Our analysis reveals convergence in two main narratives and underlying framings that diverge in the causal agent mechanism and problem- and solution-framing and show asymmetries amongst local actors in terms of their ability to participate, ability to maintain participation, and their adaptation strategies in view of changing external factors.

Large producers and exporters and their representatives are mainly worried about an increasing multitude of sustainability standards that are not seen as beneficial but rather reflect a democratic deficit at the international level, combined with retailers’ low-price policies and aggressive pricing strategies. Smallholder producers are mainly worried about their challenges in sustaining small-scale farming due to competitiveness, productivity, commercialization problems, widespread disrespect, and a lack of enforcement of the ‘banana law’, leading to a loss of protection for smallholder producers. This group of actors has historically benefited from the organic and Fairtrade market segment; however, much room has been lost since large plantations captured this lucrative market. Strategies have been taken by actors in response to the increasing market power of downstream actors in the main traditional markets; for example, organisation in a cluster to denounce unfair trading practices, and building alternative trading routes to less stringent markets. We link these narratives and underlying framings to existing literature and discursive politics to contribute to social transparency and address the democratic deficit in agrifood value chains. The concerted actions of a diverse set of stakeholders must be harnessed to address the disconnect between producing and consuming countries, driven by dialogue ‘socialised’ from production to the consumption end of the value chain, which coherent policy agendas can support. A number of initiatives, such as the European Farm to Fork strategy, offer producer and consumer countries the opportunity to establish the supportive environment necessary for companies along international value chains to operate more sustainably. However, compatibility between standards and local realities (‘institutional fit’) should be a priority for global sustainability standards adoption. Future research needs to go beyond a focus on food cost vs. nutritional value and include relations of shared responsibility across the value chain, from producers to consumers, and the perception of value chain actors in remote countries.

## Footnotes

1 Outline titled “Ayuda memoria de la Producción Orgánica Total y del Banano en el Ecuador”. Unpublished [in Spanish] provided by the National Phyto and Zoosanitary Regulation and Control Agency (Organic Direction).

2 Outline titled “Ayuda memoria de la Producción Orgánica Total y del Banano en el Ecuador”. Unpublished [in Spanish] provided by the National Phyto and Zoosanitary Regulation and Control Agency (Organic Direction).

3 The terms ‘discussed’ and ‘socialised’ refer to the process of effective participation in standard setting and the level of acceptance of the resulting regulation.

4 In 1988, European solidarity groups began certifying Fair Trade coffee. From this, Fair Trade certification programmes emerged based on established Fair Trade principles (see Raynolds 2017). In 1997, several labelling organisations established Fairtrade Labelling Organizations International (FLO), now called Fairtrade International (see Harris 2021). Driven by major international chains that sought Fair Trade certification, FLO imposed a temporary moratorium on certifying Latin American plantations and reviewed its labour standards in 2005 (Harris 2021). In September 2011, after years of discussion over whether the Fair Trade label should only be available to small-scale producers, Fair Trade USA separated from FLO (Dragusanu 2014; Raynolds 2017). Although Fair Trade bananas were introduced in Europe as far back as 1996, over the past decade, private plantations have entered the Fair Trade banana market (Moore 2004; Murray and Raynolds 2000; Smith 2010; Jaffee and Howard 2010; Harris 2021). Currently, a number of Fair Trade labels with slightly different visions are offered to consumers.

5 This refers to inequitable land distribution, with a heavy concentration of ownership and control over land and water resources since colonial times.
